# Enhancing Functional Compounds in Sesame Oil through Acid-Soaking and Microwave-Heating of Sesame Seeds

**DOI:** 10.3390/foods13182891

**Published:** 2024-09-12

**Authors:** Jitkunya Yuenyong, Suchintana Limkoey, Chonlathit Phuksuk, Thitima Winan, Chonlada Bennett, Sudarat Jiamyangyuen, Sugunya Mahatheeranont, Phumon Sookwong

**Affiliations:** 1 Rice and Cereal Chemistry Research Laboratory, Department of Chemistry, Faculty of Science, Chiang Mai University, Chiang Mai 50200, Thailand; jitkunya_y@cmu.ac.th (J.Y.); suchintana_limkoey@cmu.ac.th (S.L.); chollatis12@gmail.com (C.P.); thitima.winan7@gmail.com (T.W.); chonlada.b@cmu.ac.th (C.B.); sugunya.m@cmu.ac.th (S.M.); 2 The Graduate School, Chiang Mai University, Chiang Mai 50200, Thailand; 3 Material Science Research Center, Faculty of Science, Chiang Mai University, Chiang Mai 50200, Thailand; 4 Division of Food Science and Technology, Faculty of Agro-Industry, Chiang Mai University, Chiang Mai 50100, Thailand; sudarat.j@cmu.ac.th; 5 Center of Excellence for Innovation in Chemistry, Faculty of Science, Chiang Mai University, Chiang Mai 50200, Thailand; 6 The Functional Food Research Center for Well-Being, Multidisciplinary Research Institute, Chiang Mai University, Chiang Mai 50200, Thailand

**Keywords:** acid-soaking, microwave-heating, pre-treatment, sesamin, sesamolin, tocopherol, phytochemicals, oxidative stability, antioxidant activity, sesame

## Abstract

This study investigated whether pre-treating sesame (*Sesamum indicum* L.) seeds with a combination of acid-soaking and microwave-heating could significantly enhance the quality of the resulting sesame oil, particularly by increasing its content of functional compounds such as lignans, tocopherol, phytosterol, and squalene. The study revealed that soaking the sesame seeds in a solution of HCl and citric acid, along with microwave-heating, significantly increased the content of these compounds. The detected ranges were sesamin (1365–6927 µg g^−1^), sesamolin (605–3493 µg g^−1^), tocopherol (69.31–282.76 µg g^−1^), asarinin (ND–383.52 µg g^−1^), sesamol (ND–49.59 µg g^−1^), phytosterol (3690–6201 µg g^−1^), and squalene (532−1628 µg g^−1^). Additionally, the study found that the pre-treatment of sesame seeds had a minimal effect on the fatty acid composition, antioxidant activity (92.94–95.08% DPPH scavenging activity), and oxidative stability (2.13–2.90 mg MDA kg^−1^ oil). This is the first study to demonstrate that using acid-soaking and microwave-heating to prepare sesame seeds can produce sesame oil enriched with functional compounds, potentially benefiting cosmetic, pharmaceutical, and health applications.

## 1. Introduction

Sesame oil, an oil from sesame seeds (*Sesamum indicum* L.), is famous as a treasure trove of nutritional and medicinal benefits. Its rich nutritional profile is attributed in part to its bioactive lignans and tocopherols. Sesamin and sesamolin are predominant lignans in sesame oil, possessing health-promoting effects, including antioxidant, anti-inflammatory, anti-cancer, and cardioprotective properties, and they can improve metabolic health [1,2]. Additionally, tocopherols have antioxidant properties against oxidative stress and related chronic diseases, including cardiovascular diseases and cancer. Tocopherols can also contribute to skin health, neuroprotection, and immune system support [3,4].

The quantity and quality of sesame oil can be largely affected by the pre-treatment of sesame seeds before oil extraction. Various pre-treatment methods, such as roasting and drying, play a significant role in altering the physical and chemical properties of seeds, thereby facilitating the extraction process [5]. For instance, roasting is the most frequently used pre-treatment technique that induces several transformations of the seeds, such as removing moisture, deactivating enzymes, and mobilizing lipids. These changes enhance the flavor and aroma of the extracted oil, making it more appealing for cooking. Oven drying is another pre-treatment method commonly used to reduce the moisture content of seeds before extraction. It promotes the breakdown of cell walls and enhances oil extraction efficiency by increasing the accessibility of oil-containing cells to extraction solvents [6]. Recently, microwave treatment has been applied for the rapid heating of sesame seeds, leading to thermal decomposition or the rearrangement of sesamin precursors. Furthermore, microwave treatment may activate endogenous enzymes, such as lipases or glucosidases, which can hydrolyze sesamin precursors and release free sesamin molecules, contributing to the overall increase in sesamin content [7,8]. From this background, the pre-thermal treatment of sesame seeds plays a vital role in the preparation of sesame oil with a desired oil yield and quality.

On the other hand, the use of acids is widespread in various food applications. The primary role of acid-soaking in food processing is to alter taste or extend shelf life. While acids are generally safe, improper use or handling can lead to the formation of potentially hazardous compounds like acrylamide, especially in starchy foods during high- temperature processing [9]. Recently, a study by Chen et al. demonstrated that in vinegar, sesame lignans (sesamin and sesamolin) could be acidolyzed and changed to another structure of lignan (asarinin and sesamol, respectively) [10], corresponding to the concept of epimerization. The epimerization of sesamin involves changes at the chiral center near the methylenedioxy bridge, while sesamolin’s epimerization is influenced by an additional methoxy group affecting its stereochemistry [11]. Asarinin has been reported to possess stronger anti-inflammatory activity as it can inhibit the activation of nuclear-factor-kappa B (NF-κB), thereby reducing inflammation more effectively than sesamin [12,13]. Moreover, asarinin has additional health properties, such as immunosuppression and anti-tumor effects [14,15]. In contrast, sesamol is the primary metabolite of sesamolin with several bioactive properties, including cardioprotective and neuroprotective effects [16]. The acidolysis involves the reaction between triglycerides and free fatty acids in the presence of an acid catalyst. The chemical process involving the exchange of functional groups under acidic conditions holds the potential to modify and influence the levels of sesamin and sesamolin in sesame oil [17].

Based on the previously discussed background (in the second and third paragraphs), it is feasible to apply both pre-thermal treatment and acid-soaking to enhance the quality of sesame oil. Given that this combined method has not been previously investigated, this study aimed to study the influence of microwave treatment and acid-soaking with different kinds of acid solution on the oil quality (such as the content of lignans, tocopherol, phytosterol, and squalene). Additionally, the stability of the oil was assessed through its fatty acid composition, antioxidant activity, and oxidative stability to ensure that sesame oil prepared from the pre-treated sesame seeds had a minimal negative health effect. The obtained knowledge would be useful for the preparation of superior-quality sesame oil applicable for cosmetic, pharmaceutical, and health purposes.

## 2. Materials and Methods

### 2.1. Oilseed Samples

Black sesame seeds (*Sesamum indicum* L.) were purchased from a local farmer in the Pang Mapha district, Mae Hong Son province, Thailand (longitude, 98.1994276° E and latitude, 19.5962264° N). The used sesame variety was Mae Hong Son black sesame, a landrace variety, which is one of the most popular sesame varieties cultivated in Thailand. The harvest period of the seeds was in September 2023. The seeds were vacuum-packed and stored at room temperature. Prior to the experiment, the moisture content of the sesame samples was measured at room temperature using a grain moisture tester (Riceter f520, Kett, Japan) and was found to be below 7%.

### 2.2. Chemicals

Standard sesamin (PubChem CID: 72307, purity ≥ 98%) and sesamolin (PubChem CID: 101746, purity ≥ 98%) were purchased from Biopurity Phytochemicals Ltd. (Chengdu, China). Sesamol (PubChem CID: 68289, purity ≥ 97%) was purchased from Sigma Aldrich Corp., Ltd. (St. Louis, MO, USA). L-Asarinin (PubChem CID: 1869417, purity ≥ 98%) was purchased from Toronto Research Chemicals (North York, ON, Canada). Standard α-, β-, γ-, and δ-tocopherols (PubChem CID: 14985, 6857447, 92729, and 92094, respectively, purity ≥ 95%) were purchased from Eisai Food & Chemical Co., Ltd. (Tokyo, Japan). Standard phytosterols (PubChem CID: 87575667, purity ≥ 95%) and squalene (PubChem CID: 637072, purity ≥ 98%) were purchased from Tokyo Chemical Industry Co., Ltd. (Tokyo, Japan). 1,1-Diphenyl-2-picrylhydrazyl (DPPH, PubChem CID: 2735032, purity ≥ 97%) and thiobarbituric acid (TBA, PubChem CID: 2723628, purity ≥ 98%) from Sigma Aldrich Corp., Ltd. (St. Louis, MO, USA). HPLC-grade solvents, including n-hexane (PubChem CID: 8058), tetrahydrofuran (PubChem CID: 8028), acetonitrile (PubChem CID: 6342), and 2-propanol (PubChem CID: 3776) were purchased from RCI Labscan Co., Ltd. (Bangkok, Thailand). Analytical reagent grade hydrochloric acid 37% (HCl, PubChem CID: 313), citric acid monohydrate (PubChem CID: 22230), dichloromethane 99.5% (PubChem CID: 6344), glacial acetic acid (PubChem CID: 176), and toluene (PubChem CID: 1140) were from QReC™ Co., Ltd. (New Zealand).

### 2.3. Acid-Soaking and Microwave-Treatment

**Figure 1:**
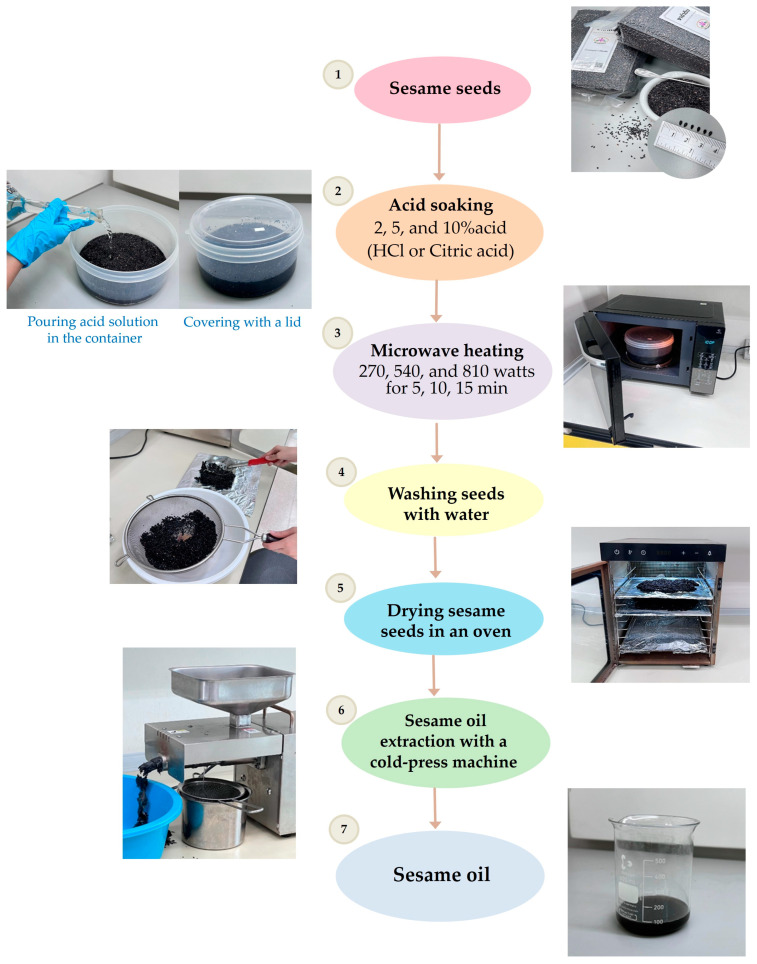
Diagram for experimentation including acid-soaking, microwave treatment, and sesame oil extraction.

The overall experimental flow is presented in Figure 1, which includes acid-soaking, microwave-heating, and sesame oil extraction.

The first part of the experiment (result in Section 3.1.1, Section 3.1.2 and Section 3.1.3) was to observe the individual influence of acid-soaking or microwave-heating on the quality of the sesame oil. Considering the influence of acids, sesame seeds (300 g) were soaked with a hydrochloric acid solution (1000 mL, at concentrations of 2, 5, and 10%, *v*/*v*) for 30 min in a plastic container without microwave-heating. For the influence of the heating, the plastic container filled with sesame seeds (300 g) and deionized water (1000 mL) was placed in the middle of the rotary plate of the microwave oven (R-652PBK, cavity of 44.00 × 25.80 × 35.70 cm, Sharp, Thailand) at operation powers of 180, 540, and 900 watts for 30 min. Then, the selection of acids (hydrochloric acid (HCl), sulfuric acid, citric acid, and phosphoric acid) was performed with a concentration of 5%, a microwave power of 540 watts, and an exposure duration of 10 min.

The second part of the experiment (result in Section 3.1.4) was to investigate whether both acid-soaking and microwave-heating could affect the quality of the sesame oil. A series of experimental trials was designed using a Box–Behnken design (BBD) from Minitab software (version 21.4.1). BBD is an experimental design in response surface methodology (RSM) used to optimize processes by exploring quadratic response surfaces. It tests variable interactions at the midpoints of edges and the center of a multidimensional box, avoiding extreme conditions. In this study, BBD considered three parameters: *A*, acid concentration (2, 6, and 10%); *B*, microwave power (270, 540, and 810 watts); and *C*, heating time (5, 10, and 15 min) that significantly affected the content of lignans (*Y*_1_) and tocopherol (*Y*_2_). The BBD was used in this study due to its fewer experimental runs, better prediction within the interior of the design space, simplicity in the experimental setup, and rotatability and orthogonality compared to other methods, such as a central composite design [18]. With the method, a total of fifteen experimental runs with three center points were designed. The runs were randomized, and the details of each experimental condition are provided in Table 1 and Table 2. All analyses for each experimental run were performed in triplicate.

In this study, control groups refer to sesame seeds untreated with acid-soaking and microwave-heating.

### 2.4. Sesame Oil Extraction

After acid-soaking and heating, sesame seeds were thoroughly dried using a food dehydrator (SS-10H, cavity of 31.5 × 39 × 41.5 cm, China) at a temperature of 70 °C for 45 min. Sesame oil extraction was performed with a home automatic oil press machine (1500 W, General Equipment Co., Ltd. (Guangzhou, China)). The cold extraction occurred at a temperature of 40–50 °C, and the sample mass of each extraction in this study was 300 g of each plant material.

### 2.5. Analysis of Lignans, Tocopherol, Phytosterol and Squalene

Analysis of those compounds was based on our previously developed high- performance liquid chromatography coupled with a diode array and fluorescence detector (HPLC–DAD–FLD) method [19]. Briefly, a Vertisep™ UPS silica HPLC column (4.6 × 250 mm, 5 µm, Vertical Chromatography Co., Ltd., Bangkok, Thailand) and the mobile phase consisting of n-hexane, tetrahydrofuran, 2-propanol, and acetonitrile were utilized for the analysis at 30 °C with a flow rate of 0.8 mL min^−1^. Lignans and tocopherols were fluorescently detected with an excitation wavelength of 295 nm and an emission wavelength of 330 nm. A diode-array detector was used for the quantification of phytosterols and squalene at 210 nm. The concentrations of the targeted compounds were calculated with the calibration curve of their standards, and the quantitative data were represented as µg g^−1^ of the sample.

### 2.6. Analysis of Fatty Acid Composition

Fatty acid composition in the obtained sesame oil was operated using gas chromatography-mass spectrometry (GC-MS) as previously described [20]. The oil samples were methylated to form fatty acid methyl esters (FAMEs) before analysis. An HP-5MS capillary column (30 m × 0.25 mm, 0.25 μm) was used with column temperature set at 165 °C, followed by a 4 °C min^−1^ for analysis 32 min, oven temperature ramped to 290 °C. The inlet temperature was 250 °C, the injection volume was 1.0 μL, and the carrier gas flow at 1 mL min^−1^. The ion-source temperatures were MS Quad 150 °C and MS source 230 °C, respectively. Detected components were identified by matching their mass spectra and retention times with standards in the Wiley05 Mass Spectral Library.

### 2.7. DPPH Assay

The antioxidant activity by DPPH radical scavenging assay was modified from Blois and Yamasaki et al. [21,22] Stock solutions of 1,1-diphenyl-2-picryl-hydrazyl (DPPH), 0.0039 g in 100 mL, and sesame oil samples, 1.0 xx g in 10 mL, were prepared in dichloromethane. In a reaction tube wrapped with aluminum foil, 1.00 mL of sample was reacted with 2.00 mL DPPH to obtain a total volume of 3.00 mL and kept at room temperature for 30 min in the dark. As a control, 1.00 mL of dichloromethane was used in place of the sample oil (1.00 mL). Spectrophotometric measurements were performed at 517 nm using a UV-visible spectrophotometer (Evolution 201, Thermo Scientific, Madison, WI, USA). Analysis was carried out in triplicates. The determination of DPPH activity (%) is conducted using the following equation.

DPPH activity (%) = ((Abs_control_ − Abs_sample_)/Abs_control_) × 100

### 2.8. Thiobarbituric Acid Reactive Substance (TBAR) Value

The TBAR value was determined using the methodology described previously by Zeb et al. [23] with some modifications. Sesame oil (0.04xx g) was added to 3.0 mL of glacial acetic acid in a screw cap tube. Then, 3.00 mL of TBA solution (200 mg TBA in 100 mL of glacial acetic acid) was added, and the mixture was placed in a water bath at 95 °C for 1 h. After cooling to room temperature, the intensity of coloration was measured at 532 nm using the UV-visible spectrophotometer. The values were expressed as mg malondialdehyde (MDA) kg^−1^.

### 2.9. Statistical Analysis

All quantitative data were obtained in triplicates (*n* = 3) and expressed as the mean ± standard deviation. The experimental design for BBD was conducted for RSM, and the subsequent statistical analyses, including ANOVA, contour plots, 3D surface graphs, and Pareto charts, were performed using Minitab (version 21.4.1, trial version, Minitab LLC, State College, PA, USA). The goodness of fit of the model equation was evaluated based on the significance of the overall model, which was assessed through an F-test (*p* < 0.05 indicating statistical significance). The significance of individual regression coefficients was assessed using *t*-tests (*p* < 0.05 indicating statistical significance). The experimental data were fitted to a quadratic model with a lack of fit that was statistically significant (*p* > 0.05). Significant differences were assessed by post-hoc Tukey’s test with the level of significance at 95% (*p* < 0.05).

## 3. Results and Discussion

### 3.1. The Effect of Acid-Soaking and Microwave-Heating of Sesame Seeds on Sesame Oil Quality

#### 3.1.1. The Effect of Acid-Soaking

**Figure 2:**
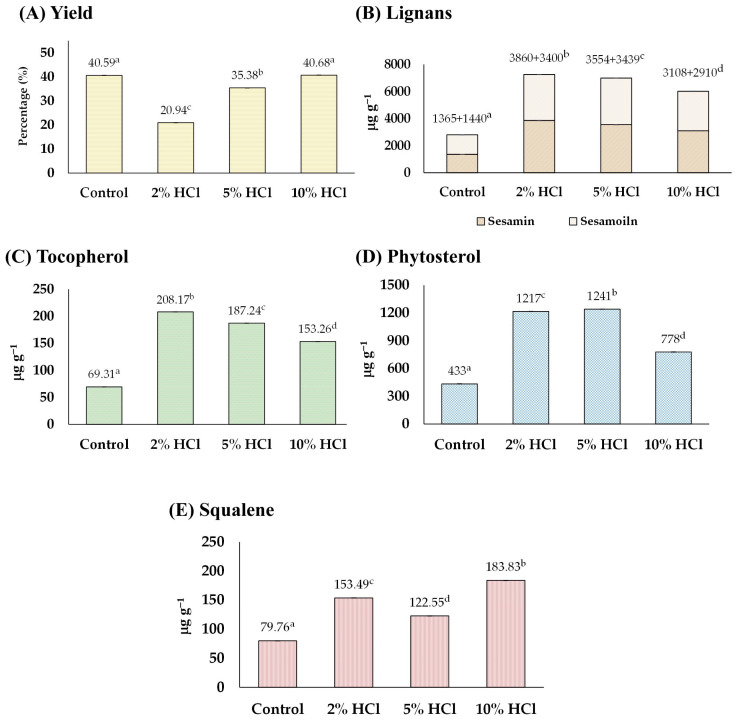
The effect of acid-soaking on (**A**) yield, and content of (**B**) lignans, (**C**) tocopherol, (**D**) phytosterol, and (**E**) squalene of the extracted sesame oil. Means denoted by different letters indicate significant differences between treatments (*p* < 0.05). Means with the same letters indicate no significant difference between the values.

Before sesame oil extraction, sesame seeds were soaked in HCl solutions (2%, 5%, and 10%) for 30 min. It was observed that soaking sesame seeds in an acid solution could soften the sesame seeds’ hardness while they remained intact but caused their color to fade. Increasing the acid concentration further softened the seeds and faded their color. The analysis, including the yield and content of tocopherol, lignans, phytosterol, and squalene obtained from the sesame seed oil, is shown in Figure 2. 

In Figure 2A, it can be observed that the oil yield decreased with 2% and 5% acid treatments from 40.59% of the control to 20.94% and 35.38%, respectively, whereas the yield of the 10% acid treatment group remained unchanged (40.68%). The reduction in the yield (2% and 5% conditions) may result from acidic conditions that cause premature oil release, degrade oil molecules, and stimulate enzymatic activities that further diminish the oil yield [24]. On the other hand, after soaking sesame seeds in acid solutions, the number of functional compounds, including lignans (sesamin and sesamolin), tocopherol, phytosterols, and squalene were all increased by up to 255, 300, 286, and 230%, respectively, compared with those of control groups (Figure 2B–E). The highest concentration of total lignan (sesamin and sesamolin) was found in the 5% group (total lignan 7260 µg g^−1^ consisting of sesamin 3860 µg g^−1^ and sesamolin 3400 µg g^−1^), tocopherol in the 2% group (208.17 µg g^−1^), phytosterols in the 5% group (1241 µg g^−1^), and squalene in the 10% group (183.83 µg g^−1^). The increase in the number of phytochemicals was partly because of the interaction of acids with lipid molecules, resulting in the formation of esters and other derivatives. This process can activate enzymes that play a role in phytochemical biosynthesis pathways. Moreover, acidic conditions can facilitate the breakdown of complex molecules into simpler, bioactive forms, thereby enhancing their concentration in the seed extract [17]. The findings were first evidenced that soaking sesame seeds in an acid solution before oil extraction could enhance the number of phytochemicals in the consequent sesame oil.

#### 3.1.2. The Effect of Microwave-Heating

**Figure 3:**
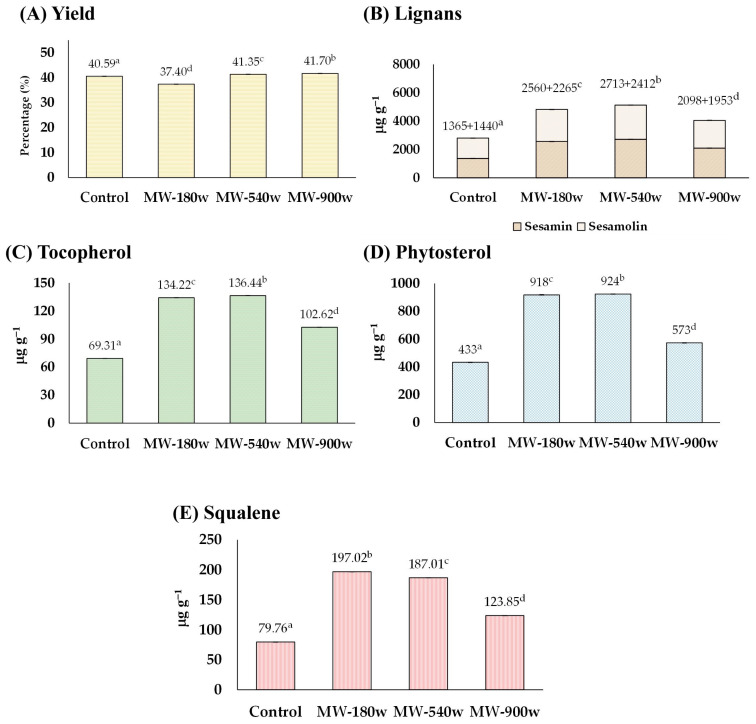
The effect of microwave-heating on (**A**) yield, and content of (**B**) lignans, (**C**) tocopherol, (**D**) phytosterol, and (**E**) squalene of the extracted sesame oil. Means denoted by different letters indicate significant differences between treatments (*p* < 0.05). Means with the same letters indicate no significant difference between the values.

The sesame seeds soaked with water were heated with microwave powers of 180, 540, and 900 watts for 30 min without the addition of acid. It was also found that microwave-heating could soften the seeds and slightly discolor them. The quality of the sesame oil derived from the pre-microwave heated seeds is exhibited in Figure 3. The yield percentage of sesame oil from sesame seeds was comparable to that of the control (Figure 3A). The amounts of lignans, tocopherol, phytosterols, and squalene were all enhanced up to 181, 198, 213, and 247%, respectively, compared with those of the control groups (Figure 3B–E). The highest concentration of lignan was found at 540 watts (total lignan 5125 µg g^−1^ consisting of sesamin 2713 µg g^−1^ and sesamolin 2412 µg g^−1^), tocopherol at 540 watts (136.44 µg g^−1^), phytosterols at 540 watts (924 µg g^−1^), and squalene at 180 watts (197.02 µg g^−1^).

This result was consistent with previous research suggesting that the microwave treatment of sesame seeds could significantly impact the number of bioactive compounds in the sesame oil [25,26]. Thermal treatment of sesame seeds or oil enhances sesamin and sesamolin availability by breaking down complex molecules. It increases their solubility, aiding extraction. Microwave-heating can alter the nutritional composition, affecting vitamins, minerals, bioactive compounds, lipid oxidation rates, fatty acid profiles, and antioxidant levels in sesame oil, thus impacting its overall stability and health benefits [27,28].

#### 3.1.3. The Selection of Acid Solution

Based on the results of Figure 2 and Figure 3, it was possible to combine the use of acid soaking and microwave-heating to improve the content of functional compounds in sesame oil. Accordingly, it was necessary to find a suitable acid for acid soaking. Two strong acids (HCl and sulfuric acid) and two weak acids (citric acid and phosphoric acid) applicable in food processing were considered in this part of the study. The type of acid would be chosen based on the content of lignans and tocopherol, which are characteristic compounds in sesame oil.

**Figure 4:**
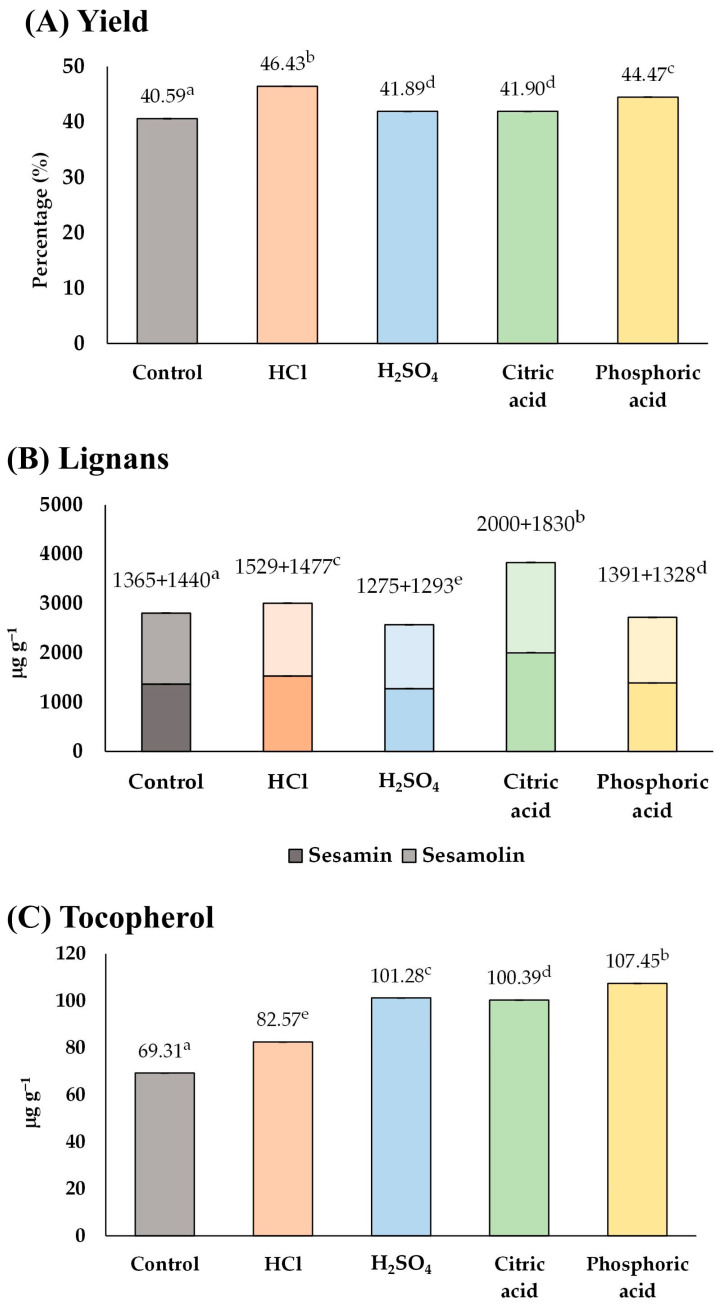
The influence of type of acid solution on (**A**) yield, and content of (**B**) lignans, and (**C**) tocopherol of the extracted sesame oil. Means denoted by different letters indicate significant differences between treatments (*p* < 0.05). Means with the same letters indicate no significant difference between the values.

As a result (Figure 4), the control group contained 2805 µg g^−1^ of total lignan and 69.31 µg g^−1^ of tocopherol. Among the groups treated with acid, HCl and citric acid appeared to be the suitable acids for soaking sesame seeds because they could enhance both lignans (3006 and 3830 µg g^−1^) and tocopherol (82.57 and 100.39 µg g^−1^) in the sesame oil, respectively. In contrast, soaking sesame seeds in sulfuric acid and phosphoric acid solutions could improve the tocopherol content (101.28 and 107.45 µg g^−1^) but lower the lignan content (2568 and 2719 µg g^−1^), respectively. Therefore, HCl and citric acid were chosen as the most suitable acids that would be used in the consequent experiments (Section 3.1.4).

#### 3.1.4. The Combined Effect of Acid-Soaking and Microwave-Heating 

In this part of the study, we studied the influence of acid-soaking (strong acid HCl or weak acid citric acid) in conjunction with microwave-heating as a pre-treatment method of sesame seeds prior to sesame oil extraction. The yield of oil and the content of lignan, tocopherol, phytosterol, and squalene are exhibited in Table 1 and Table 2 for HCl and citric acid treatments, respectively. The results from those treatments were compared with the results of the control sample that had 40.59% of the extraction yield, 69.31 µg g^−1^ of tocopherol (only γ-tocopherol isoform found in sesame oil), 2805 µg g^−1^ of the total lignans (consisting of 1365 µg g^−1^ of sesamin and 1440 µg g^−1^ of sesamolin), 3690 µg g^−1^ of phytosterol, and 532 µg g^−1^ of squalene.

For treatments with the HCl solution (Table 1), the content of the target compounds ranged as follows: sesamin from 1365 to 6927 µg g^−1^, sesamolin from 606 to 3493 µg g^−1^, γ-tocopherol from 69.31 to 282.76 µg g^−1^, asarinin from ND to 383.52 µg g^−1^, sesamol from ND to 49.59 µg g^−1^, phytosterol from 3690 to 6201 µg g^−1^, and squalene from 532 to 1628 µg g^−1^. It was noted that asarinin could only be found in some conditions, including H1 (10% HCl, 540 watts, for 15 min), H13 (6% HCl, 810 watts, for 15 min), and H14 (10% HCl, 540 watts, for 15 min) at 332.56, 118.30, and 383.52 µg g^−1^, respectively. Sesamol was also limited to three conditions (H1, H13, and H14) in the range of 28.97–49.59 µg g^−1^, and in H8, H9, and H11 (6% HCl, 540 watts, for 10 min) in the range of 28.73–33.84 µg g^−1^. The presence of asarinin and sesamol could be from the acidolysis of sesamin and sesamolin under acidic and thermal conditions [10]. On the other hand, considering the treatments with the citric acid solution (Table 2), sesamin was found from 1365 to 6634 µg g^−1^, sesamolin from 1440 to 3986 µg g^−1^, γ-tocopherol from 69.31 to 314.83 µg g^−1^, phytosterol from 3690 to 6460 µg g^−1^, and squalene from 532 to 1360 µg g^−1^. It was noted that no asarinin and sesamol were found in any citric acid treatment groups, suggesting that citric acid could not influence the acidolysis of lignan in the sesame seeds. 

This observation supports the idea that both asarinin and sesamol are produced under high temperatures or acidic conditions. HCl, a strong acid, can break down sesame seed components, promoting the hydrolysis of lignans and other precursors to form these compounds [29]. In contrast, soaking seeds in citric acid, a weaker acid with a lower dissociation constant (pKa) and buffering capacity, does not generate sesamol or asarinin. The acid’s strength plays a crucial role in these reactions. However, even with HCl, only certain conditions produce sesamol and asarinin, possibly due to the sesame seed coat acting as a protective barrier against the acid, thereby reducing the acidolysis of lignan.

To optimize the operation conditions for sesame oil extraction, an experimental design using RSM was employed. The model fitting with the experimental data (Table 1 and Table 2) was performed, and the regression coefficients and the analysis of variance for the models related to lignans and tocopherol content are presented in Table 3 and Table 4. According to Table 3, for HCl treatments, the most significant independent variables for lignan content were *A* (*p*-value of 0.005) and *A*^2^ (*p*-value of 0.033), while no significant variables were found for tocopherol content.

For citric acid treatment, the significant parameter was *C*^2^ for both lignans and tocopherol content (*p*-value of 0.013 and 0.031, respectively). The regression coefficients (Table 3) enable the models, consisting of linear and quadratic terms for *A* (acid concentration), *C* (heating time), and intercept values, to be expressed by the following equations: for HCl treatment, *Y*_1_ = 7528 − 675*A* + 60.1*A*^2^; for citric acid treatment, *Y*_1_ = 16,425 + 49.6*C*^2^ and *Y*_2_ = 484 + 1.735*C*^2^. Notably, the HCl and citric acid groups exhibited different trends across the variables. Specifically, the acid concentration had a greater effect in the HCl treatments, whereas heating time had a stronger effect in the citric acid treatments.

On the other hand, the *p*-values for the overall model (ranging from 0.051 to 0.425) and the *p*-values for the lack-of-fit test (ranging from 0.140 to 0.477) across the four systems were relatively high (Table 4). The non-significant *p*-values for the model suggest that the factors included may not have a statistically significant impact on the response variable, indicating that the model structure or the chosen factors might not be capturing meaningful effects. However, the high *p*-values of the lack-of-fit test imply that the model fits the data reasonably well.

To assess uniform precision across the design space, the analysis revealed that for HCl treatments, the precision of the three center points (expressed as % relative standard deviation, RSD) and the variances across other points were 1.67 and 14.33 for the total lignans, 8.82 and 16.79 for tocopherol, 12.17 and 16.22 for phytosterol, and 6.09 and 4.44 for squalene. In comparison, citric acid treatments exhibited variances of 1.72 and 12.84 for the total lignans, 6.32 and 14.70 for tocopherol, 1.88 and 18.70 for phytosterol, and 9.09 and 24.02 for squalene. The results indicate that the BBD (Box–Behnken Design) offers better precision at the center points but less reliability at other points in the design space, with increased variability outside the center affecting prediction accuracy. Improving precision may require refining the design or increasing the number of experimental runs in high-variance areas.

**Figure 5:**
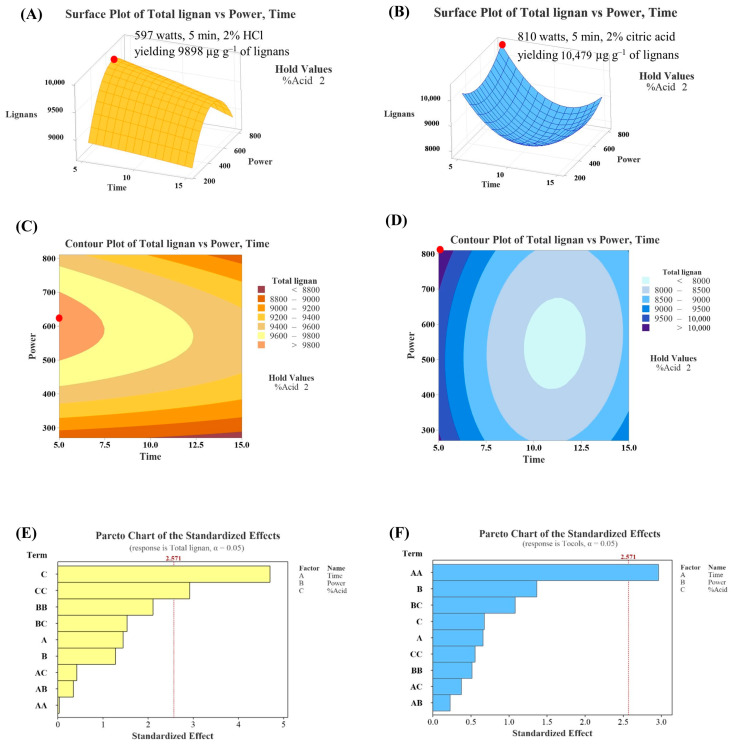
Three-dimensional response surfaces, contour plots, and Pareto charts illustrating lignans content under different pre-treatment conditions of HCl (**A**,**C**,**E**) and citric acid (**B**,**D**,**F**) systems. The red dot indicates the location of the optimum condition.

Figure 5A,B depict the three-dimensional response surfaces for total lignan content from the HCl and citric acid systems, respectively. For the HCl system, the response surface forms a horizontal plane curving downward, with the highest point located in the top-left corner of the visible plane (2% HCl solution at 597 watts for 5 min). In contrast, for the citric acid system, the plane curves upward with the highest point also located in the top-left corner (2% citric acid solution at 810 watts for 5 min). Figure 5C,D show the contour plots for total lignan content, where the highest response (the highest lignan content) is located at the left edge of the plots. The Pareto chart (Figure 5E,F) indicates that terms *C* and *C*^2^ are significant parameters in the HCl system, while term *A*^2^ is significant in the citric acid system.

Using RSM, it was predicted that sesame oil with the highest lignan content would be obtained from sesame seeds treated with a 2% HCl solution at 597 watts for 5 min, yielding 9898 µg g^−1^ of lignans (HCl system), and from sesame seeds treated with a 2% citric acid solution at 810 watts for 5 min, yielding 10,479 µg g^−1^ of lignans (citric acid system). The calculated maximum response values (tocopherols and lignans) were obtained for borderline levels of HCl concentration and time, so changes in the levels of these experimental conditions should be reconsidered in further studies. Under the predicted conditions, the actual lignan content was determined to be 6621 µg g^−1^ (2% HCl solution at 640 watts for 5 min) and 7660 µg g^−1^ (2% citric acid solution at 810 watts for 5 min). These results suggest that pre-treating sesame seeds under the predicted conditions did not result in sesame oil with the highest lignan content. This discrepancy was likely due to the lack of model significance (Table 4), implying that the selected model was inadequate to accurately predict the relationship among the evaluated parameters [30]. Typically, RSM assumes a quadratic relationship between variables, and the true relationship may not be accurate as real-world relationships could be linear, higher-order polynomial, or non-polynomial. Additionally, RSM requires attention to model validation and uncertainty quantification to ensure reliable conclusions as it is crucial to assess the uncertainty in model parameters and predictions [31].

Therefore, instead of using conditions from predictive equations from RSM, we relied on experimental data because the data gave us sesame oil with the highest content of lignans and tocopherol. The most suitable conditions for HCl treatments for sesame oil with the highest lignan, tocopherol, and phytosterol content (10,420 µg g^−1^ of lignan, 282.76 µg g^−1^ of tocopherol, and 6201 µg g^−1^ of phytosterol) were conditions (H15) utilizing 2% HCl, microwave power of 540 watts, and a heating duration for 5 min. Similarly, regarding citric acid treatment, condition C1, employing 6% acid, a power of 810 watts, and a heating time of 5 min, could supply the sesame oil with the highest lignan and tocopherol content (10,297 µg g^−1^ and 314.83 µg g^−1^, respectively), while the condition C3 (6% acid, 810 watts, for 5 min) and condition C8 (6% acid, 810 watts, for 15 min) provided oil with the highest phytosterol and squalene content, respectively.

Prior to the present study, we considered acidolysis as the decomposition of the precursor lignans (sesamin and sesamolin) into smaller lignans (asarinin and sesamol, respectively). We attempted to conduct acidolysis using acid soaking and microwave-heating. However, the occurrence of acidolyzed asarinin and sesamolin was found only in some HCl treatments (conditions H1, H8, H13, and H14). Unexpectedly, treating sesame seeds with acid soaking and microwave-heating could substantially improve the number of preexisting phytochemicals, including sesamin (up to 5 times), sesamolin (up to 2.7 times), tocopherol (up to 4.5 times), phytosterols (up to 1.7 times), and squalene (up to 2.4 times).

Acid-soaking and microwave-heating significantly enhance oil quality through distinct but complementary mechanisms. Acid-soaking is effective because it interacts with lipid molecules to form esters and derivatives, which can increase the concentration of beneficial phytochemicals. The acidic conditions also activate enzymes that are crucial for phytochemical biosynthesis, facilitating the breakdown of complex molecules into simpler, more bioactive forms. This results in higher concentrations of valuable compounds. Microwave-heating further improves oil quality by efficiently breaking down complex molecules, which enhances the availability of key compounds like sesamin and sesamolin. This method increases the solubility and extraction efficiency of these compounds, leading to higher antioxidant levels in the oil. The combined effect of these processes not only boosts the nutritional profile of the oil but also improves its stability and health benefits, making these methods highly effective for optimizing oil quality [17,27].

**Figure 6:**
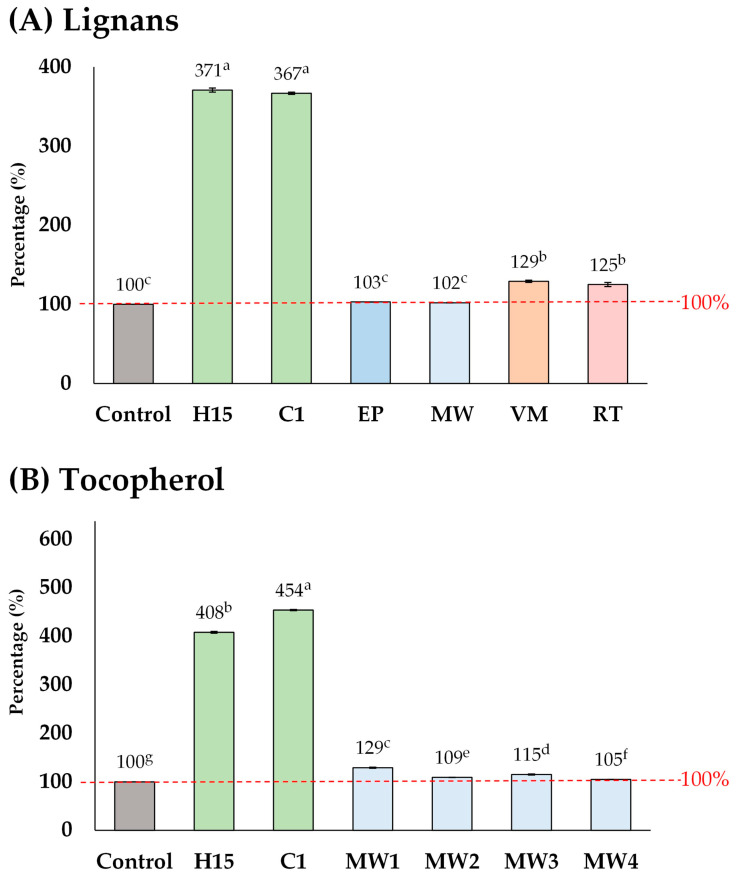
Comparison of the different pre-treatment methods of sesame seeds prior to oil extraction, including explosion-puffing (EP) [24], general microwave-heating (MW) [25,32], vacuum microwave-heating (VM) [26], and roasting (RT) [26]. Means denoted by different letters indicate significant differences between treatments (*p* < 0.05). Means with the same letters indicate no significant difference between the values.

The elevated lignan content (maximum 10,420 µg g^−1^) observed in this study exceeded the levels found in commercial sesame oil products, including supplements (1775–8965 µg g^−1^) [19]. Furthermore, the lignan contents reported here are higher than those observed in previous studies on the pre-treatment of sesame seeds prior to oil extraction, including methods such as explosion-puffing, general microwave-heating, vacuum microwave-heating, and roasting (Figure 6A) [25,26,32]. Other pre-treatment methods increased the lignan content by up to 102–129%, whereas in this study, lignan content was elevated by up to 371%. A similar trend was observed for tocopherol (Figure 6B). These results strongly suggest that acid-soaking combined with microwave-heating is a highly effective pre-treatment method for improving the phytochemical content, particularly lignan, in sesame oil.

Although the results of this study demonstrate the usefulness of acid-soaking and microwave-heating for enhancing functional compounds in sesame oil, the great diversity of sesame varieties, which differ in shape, color, and nutritional content, makes it necessary to study more than one variety to validate the effectiveness of this pre-treatment method for food and health applications. Additionally, the proposed pre-treatment method involves heating and drying steps that incur extra energy and equipment costs, which are crucial for evaluating the method’s practical application. However, these costs may be offset by the benefits, depending on the production scale and economic conditions. Therefore, further studies are needed to perform a detailed cost analysis and fully understand the implications of this method.

### 3.2. Fatty Acid Composition and Stability of the Sesame Oil

Consequently, the fatty acid composition, antioxidant activity, and oxidative stability were evaluated in selected sesame oil samples, particularly those with the highest lignan and tocol content. This selection ensures that sesame oil produced under the recommended conditions from this study would maintain high oil quality.

#### 3.2.1. Fatty Acid Composition

**Figure 7:**
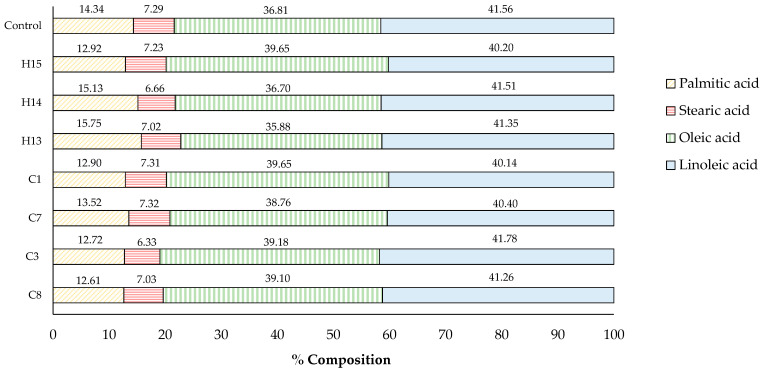
Fatty acid composition of sesame oil samples (%).

The fatty acid composition of the control sesame oil and some selected oils derived from acid-soaking and microwave-heating is shown in Figure 7. All sesame oil contained four types of fatty acids, ranging from the highest to the lowest compositional percentage: linoleic acid (C18:2, 40.14–41.78%), oleic acid (C18:1, 35.88–39.65%), palmitic acid (C16:0, 12.61–15.75%), and stearic acid (C18:0, 6.33–7.32%). It was noted that no obvious changes in the fatty acid composition of the analyzed oils were monitored, and no other fatty acid was detected using GC-MS. The findings demonstrated that acid-soaking and microwave -heating had a minimal effect on altering the fatty acid composition, thereby not deteriorating the quality of sesame oil. Similarly, the typical chemical refining process of commercial cooking oil involves using acid and heat to remove impurities and ensure the quality and stability of the final product [33,34].

#### 3.2.2. Antioxidant Activity

The antioxidant activity was evaluated using a DPPH radical scavenging assay (Table 5). The DPPH scavenging activity was 90.85% of the control group and 92.94–95.08% of the oils from the pre-treated sesame seeds. The scavenging activity of the oils from pre-treated seeds was higher than that of the control because of the greater number of bioactive compounds in the sesame oil (Table 5). A study by Ramroudi et al. found that the antioxidant activity of sesame oil was higher than other oils commonly consumed for antioxidant purposes [27]. The results (Table 5) implied that soaking the sesame seeds in an acid solution with microwave-heating before oil extraction can enhance the antioxidant activity of sesame oil suitable for various health and industrial applications.

#### 3.2.3. Oxidative Stability

Preparation of sesame seeds by acid-soaking and microwave-heating may potentially lead to oxidation, but it also enhances the presence of antioxidants. Accordingly, TBAR values were examined for their oxidative stability (Table 5). The TBAR value of the control was 2.42 mg MDA kg^−1^ oil, while those of the experimental oils were 2.13–2.90 mg MDA kg^−1^ oil. The determined TBAR values were found to be comparable to the control group and in normal ranges of general edible oils. For instance, Ansorena et al. measured TBARs around 1.11–7.57 mg MDA kg^−1^ for coconut, rapeseed, and grape seed oils [35]. A study by Ramroudi et al. found that sesame oil had the lowest TBAR values (0.91–3.24 mg MDA kg^−1^) among various edible oils (coconut, rapeseed, and grape seed), underscoring its superior oxidative stability. Sesamol and sesamin, in particular, are potent antioxidants that scavenge free radicals and protect the oil from oxidative damage [28]. Therefore, sesame oil derived from the treated sesame seeds is deemed safe and suitable for use.

Since our pre-treatment method involved both the addition of an acid solution and microwave-heating, potential drawbacks had to be considered. For the acid application (specifically HCl and citric acid in our study), both are generally safe and can be rinsed away with water. The body can manage small amounts of chloride ions from HCl without adverse effects, and the levels of citric acid applied are typically not harmful. With respect to microwave-heating, some sensitive nutrients might degrade due to high temperatures and prolonged exposure. Additionally, the heat generated during microwave processing may promote fat oxidation. However, no significant changes were observed in the fatty acid composition (Figure 7), antioxidant activity, or oxidative stability of the experimental oil (Table 5). This indicates that the conditions used were not severe enough to negatively impact the sesame oil.

## 4. Conclusions

This study evaluated the influence of acid-soaking and microwave-heating sesame seeds prior to oil extraction on the quality of the resulting sesame oil. In the first part of the experiment, the individual effects of acid-soaking and microwave-heating on oil quality were observed, showing an increase in phytochemical content even when considered separately. The second part of the experiment investigated the combined effects of acid-soaking and microwave-heating on oil quality using a BBD model. The overall results from the BBD indicated a positive influence on the phytochemical content in the extracted oil, with lignan content increasing by up to 371%, tocopherol by up to 454%, phytosterol by up to 168%, and squalene by up to 224%. While RSM provided regression equations for surface response, the predicted conditions did not yield the best results upon validation, likely due to a lack of model significance. The optimal conditions identified in this study were soaking sesame seeds in a 2% HCl solution at 540 watts for 5 min and in a 6% citric acid solution at 810 watts for 5 min, resulting in 10,420 and 10,297 µg g^−1^ of lignan, respectively. Additionally, no adverse effects were observed on the fatty acid composition, antioxidant activity (DPPH), or oxidative stability (TBARs) of the oils, suggesting the safety of the products. Although this study lays a strong foundation for using acid-soaking and microwave-heating as effective pre-treatment methods for sesame seeds prior to oil extraction, certain limitations require further consideration. These include the diversity of sesame varieties, potential variability in seed quality, alternative heating methods, and the scalability of the pre-treatment for practical food and health applications.

## Figures and Tables

**Table 1:** The content of target bioactive compounds in sesame oil extracted from sesame seeds treated with HCl soaking and microwave-heating (µg g^−1^).

Sample (Variable Codes)	%Yield	Sesamin	Sesamolin	Asarinin	Sesamol	Total Lignans (*Y*_1_)	Tocopherol (*Y*_2_)	Phytosterol	Squalene
Control	40.59 ± 0.04 ^d^	1365 ± 1.00 ^a^	1440 ± 1.53 ^d^	ND	ND	2805 ± 0.87 ^a^	69.31 ± 0.03 ^a^	3690 ± 1.53 ^a^	532 ± 0.06 ^a^
H1 [1, 0, 1] (10%: 540 W: 15 min)	48.59 ± 0.02 ^l^	5089 ± 1.03 ^e^	1121 ± 1.56 ^c^	322.56 ± 2.00 ^b^	28.97 ± 0.02 ^a^	6562 ± 0.93 ^c^	209.89 ± 0.25 ^c^	4300 ± 2.03 ^e^	750 ± 0.03 ^h^
H2 [0, −1, −1] (6%: 270 W: 5 min)	32.17 ± 0.35 ^a^	4595 ± 1.24 ^b^	2490 ± 1.51 ^i^	ND	ND	7085 ± 1.30 ^f^	167.58 ± 0.29 ^b^	4929 ± 1.25 ^j^	716 ± 0.02 ^e^
H3 [−1, 1, 0] (2%: 810 W: 10 min)	46.97 ± 0.20 ^i,j^	6022 ± 1.25 ^k^	2853 ± 1.66 ^k^	ND	ND	8875 ± 1.37 ^n^	239.13 ± 0.31 ^e,f^	4704 ± 1.26 ^g^	775 ± 0.04 ^i^
H4 [0, 1, −1] (6%: 810 W: 5 min)	39.70 ± 0.14 ^c,d^	4786 ± 1.13 ^c^	2088 ± 2.01 ^e^	ND	ND	6874 ± 1.09 ^e^	167.55 ± 0.12 ^b^	5126 ± 1.39 ^k^	737 ± 0.05 ^g^
H5 [1, −1, 0] (10%: 270 W: 10 min)	46.01 ± 0.24 ^i^	5411 ± 1.35 ^g^	2562 ± 1.57 ^j^	ND	ND	7973 ± 1.05 ^i^	214.78 ± 0.25 ^c,d^	5307 ± 1.27 ^m^	729 ± 0.03 ^f,g^
H6 [−1, −1, 0] (2%: 270 W: 10 min)	48.14 ± 0.13 ^l^	5725 ± 1.26 ^j^	2895 ± 1.23 ^l^	ND	ND	8620 ± 0.87 ^m^	212.74 ± 0.52 ^c,d^	4617 ± 1.71 ^f^	704 ± 0.04 ^d^
H7 [−1, 0, 1] (2%: 540 W: 15 min)	46.91 ± 0.25 ^i,j^	6267 ± 1.40 ^m^	3145 ± 1.58 ^m^	ND	ND	9412 ± 1.08 ^o^	240.58 ± 0.14 ^f^	3889 ± 1.64 ^d^	1363 ± 0.02 ^l^
H8 [0, 0, 0] (6%: 540 W: 10 min)	47.45 ± 0.28 ^k^	5682 ± 1.25 ^i^	2350 ± 1.62 ^g^	ND	33.48 ± 0.03 ^b^	8066 ± 1.05 ^k^	219.90 ± 0.23 ^cde^	4814 ± 1.13 ^i^	724 ± 0.06 ^f^
H9 [0, 0, 0] (6%: 540 W: 10 min)	43.50 ± 0.25 ^e,f^	5628 ± 1.33 ^i^	2369 ± 1.64 ^g^	ND	33.84 ± 0.04 ^b^	8031 ± 1.07 ^j^	211.50 ± 0.25 ^c^	4739 ± 1.25 ^h^	689 ± 0.05 ^c^
H10 [1, 0, −1] (10%: 540 W: 5 min)	45.91 ± 0.14 ^h,i^	5611 ± 1.25 ^h^	2489 ± 1.71 ^i^	ND	ND	8100 ± 1.48 ^l^	216.65 ± 0.36 ^c,d^	3700 ± 1.15 ^b^	680 ± 0.03 ^c^
H11 [0, 0, 0] (6%: 540 W: 10 min)	47.49 ± 0.25 ^k^	5677 ± 1.24 ^i^	2112 ± 1.62 ^f^	ND	30.73 ± 0.02 ^a,b^	7820 ± 1.43 ^h^	185.17 ± 0.25 ^b^	3838 ± 1.36 ^c^	641 ± 0.04 ^b^
H12 [0, −1, 1] (6%: 270 W: 15 min)	36.39 ± 0.22 ^b^	4886 ± 1.15 ^d^	2396 ± 1.72 ^h^	ND	ND	7282 ± 1.44 ^g^	169.81 ± 0.34 ^b^	3836 ± 1.35 ^c^	707 ± 0.07 ^d^
H13 [0, 1, 1] (6%: 810 W: 15 min)	45.73 ± 0.13 ^g,h^	6174 ± 1.25 ^l^	297 ± 1.61 ^a^	118.30 ± 1.53 ^a^	47.90 ± 0.03 ^c^	6637 ± 1.10 ^d^	272.71 ± 0.41 ^g^	5657 ± 1.43 ^n^	1628 ± 0.03 ^m^
H14 [1, 1, 0] (10%: 810 W: 10 min)	48.18 ± 0.15 ^l^	5251 ± 1.35 ^f^	606 ± 1.56 ^b^	383.52 ± 1.53 ^c^	49.59 ± 0.02 ^d^	6290 ± 1.12 ^b^	213.71 ± 0.21 ^c,d^	5197 ± 1.26 ^l^	865 ± 0.02 ^j^
H15 [−1, 0, −1] (2%: 540 W: 5 min)	44.50 ± 0.25 ^f,g^	6927 ± 1.22 ^n^	3493 ± 1.43 ^n^	ND	ND	10420 ± 1.33 ^p^	282.76 ± 0.51 ^g^	6201 ± 1.33 ^o^	1299 ± 0.04 ^k^

ND = nondetectable. Means denoted by different letters indicate significant differences between treatments (*p* < 0.05). Means with the same letters indicate no significant difference between the values.

**Table 2:** The content of target bioactive compounds in sesame oil extracted from sesame seeds treated with citric acid-soaking and microwave-heating (µg g^−1^).

Sample (Variable Codes)	%Yield	Sesamin	Sesamolin	Asarinin	Sesamol	Total Lignans (*Y*_1_)	Tocopherol (*Y*_2_)	Phytosterol	Squalene
Control	40.59 ± 0.04 ^d,e^	1365 ± 1.00 ^a^	1440 ± 1.53 ^a^	ND	ND	2805 ± 0.86 ^a^	69.31 ± 0.03 ^a^	3690 ± 1.53 ^a^	532 ± 0.06 ^a^
C1 [0, 1, −1] (6%: 810 W: 5 min)	46.82 ± 0.02 ^g^	6311 ± 1.12 ^m,n^	3986 ± 1.42 ^p^	ND	ND	10297 ± 0.85 ^n^	314.83 ± 0.02 ^i^	5805 ± 1.36 ^e^	649 ± 0.07 ^b,c^
C2 [−1, −1, 0] (2%: 270 W: 10 min)	43.15 ± 0.03 ^e^	5793 ± 1.32 ^j^	3066 ± 1.55 ^l,m^	ND	ND	8859 ± 1.44 ^i^	268.36 ± 0.07 ^f^	5342 ± 1.24 ^d^	761 ± 0.12 ^c^
C3 [0, 1, 1] (6%: 810 W: 15 min)	37.61 ± 0.06 ^c^	6634 ± 1.25 ^o^	3382 ± 1.23 ^o^	ND	ND	10016 ± 0.85 ^m^	287.17 ± 0.05 ^h^	6094 ± 1.35 ^f^	1252 ± 0.06 ^h^
C4 [0, −1, 1] (6%: 270 W: 15 min)	34.44 ± 0.10 ^b^	6064 ± 1.34 ^l^	2976 ± 1.75 ^k^	ND	ND	9040 ± 1.55 ^j^	228.03 ± 0.04 ^d,e^	4948 ± 1.65 ^c^	789 ± 0.04 ^d^
C5 [1, −1, 0] (10%: 270 W: 10 min)	44.66 ± 0.04 ^f^	5246 ± 1.14 ^e,f^	2824 ± 1.13 ^e,f^	ND	ND	8070 ± 1.14 ^d^	208.85 ± 0.02 ^b,c^	5475 ± 1.41 ^d^	756 ± 0.09 ^c^
C6 [−1, 0, 1] (2%: 540 W: 15 min)	29.28 ± 0.03 ^a^	5447 ± 1.35 ^h^	3139 ± 1.65 ^n^	ND	ND	8586 ± 1.50 ^g^	259.94 ± 0.03 ^e,f^	4600 ± 1.56 ^b^	819 ± 0.05 ^e^
C7 [1, 1, 0] (10%: 810 W: 10 min)	44.26 ± 0.12 ^e,f^	5389 ± 1.25 ^g,h^	2836 ± 1.24 ^f^	ND	ND	8225 ± 1.25 ^e^	227.99 ± 0.08 ^d,e^	8473 ± 1.33 ^h^	785 ± 0.04 ^d^
C8 [−1, 0, −1] (2%: 540 W: 5 min)	44.73 ± 0.14 ^f^	6379 ± 1.15 ^n^	3457 ± 1.34 ^o,p^	ND	ND	9836 ± 1.25 ^l^	275.41 ± 0.03 ^g,h^	4750 ± 2.03 ^b,c^	1360 ± 0.08 ^i^
C9 [0, 0, 0] (6%: 540 W: 10 min)	47.85 ± 0.06 ^h^	4475 ± 1.36 ^b,c^	2807 ± 1.54 ^d,e^	ND	ND	7282 ± 1.45 ^b,c^	201.58 ± 0.02 ^b^	5166 ± 1.47 ^d^	744 ± 0.10 ^c^
C10 [−1, 1, 0] (2%: 810 W: 10 min)	38.92 ± 0.11 ^d^	5272 ± 1.10 ^f^	2788 ± 1.62 ^c^	ND	ND	8060 ± 0.86 ^d^	226.72 ± 0.04 ^d^	6460 ± 1.35 ^g^	791 ± 0.04 ^d,e^
C11 [1, 0, 1] (10%: 540 W: 15 min)	39.11 ± 0.04 ^d^	6038 ± 1.37 ^k,l^	3087 ± 1.74 ^m^	ND	ND	9125 ± 1.56 ^k^	272.74 ± 0.06 ^g,h^	6139 ± 1.87 ^f^	851 ± 0.06 ^f^
C12 [0, 0, 0] (6%: 540 W: 10 min)	47.87 ± 0.05 ^h^	4305 ± 1.28 ^b^	2961 ± 1.24 ^j,k^	ND	ND	7266 ± 1.26 ^b^	223.77 ± 0.05 ^c,d^	5222 ± 1.36 ^d^	878 ± 0.05 ^g^
C13 [0, 0, 0] (6%: 540 W: 10 min)	47.85 ± 0.04 ^h^	4808 ± 1.36 ^c^	2684 ± 1.67 ^b^	ND	ND	7492 ± 1.52 ^c^	226.68 ± 0.04 ^d^	5358 ± 1.69 ^d^	764 ± 0.07 ^c^
C14 [1, 0, −1] (10%: 540 W: 5 min)	47.79 ± 0.21 ^h^	5091 ± 1.24 ^d^	3262 ± 2.03 ^n,o^	ND	ND	8353 ± 1.64 ^f^	267.19 ± 0.03 ^f^	4658 ± 1.25 ^b^	850 ± 0.10 ^f^
C15 [0, −1, −1] (6%: 270 W: 5 min)	37.07 ± 0.14 ^c^	5683 ± 1.23 ^i^	2923 ± 2.14 ^i,j^	ND	ND	8606 ± 1.69 ^h^	242.93 ± 0.06 ^e^	4947 ± 1.42 ^c^	818 ± 0.03 ^e^

ND = nondetectable. Means denoted by different letters indicate significant differences between treatments (*p* < 0.05). Means with the same letters indicate no significant difference between the values.

**Table 3:** Regression coefficients for the model predicting lignans and tocopherol content under HCl and citric acid treatments.

Term	HCl Treatment						Citric Acid Treatment					
	Lignans			Tocopherol			Lignans			Tocopherol		
	Coef	*T*-Value	*p*-Value	Coef	*T*-Value	*p*-Value	Coef	*T*-Value	*p*-Value	Coef	*T*-Value	*p*-Value
Constant	7673	21.03	0.000	205.5	11.11	0.000	7680	21.19	0.000	217.3	13.39	0.000
%Acid (*A*)	−1050	−4.70	0.005	−15.0	−1.33	0.242	−196	−0.88	0.417	−6.71	−0.67	0.530
Power (*B*)	−286	−1.28	0.257	16.0	1.41	0.217	253	1.14	0.306	13.57	1.37	0.230
Time (*C*)	−323	−1.45	0.208	7.3	0.64	0.548	−40	−0.18	0.863	−6.56	−0.66	0.538
%Acid × %Acid (*A*^2^)	961	2.92	0.033	28.8	1.73	0.145	54	0.17	0.874	8.1	0.55	0.603
Power × Power (*B*^2^)	−694	−2.11	0.089	−14.2	−0.85	0.432	569	1.74	0.142	7.5	0.51	0.629
Time × Time (*C*^2^)	−10	−0.03	0.978	3.1	0.19	0.858	1240	3.80	0.013	43.4	2.96	0.031
%Acid × Power (*AB*)	−485	−1.53	0.186	−6.9	−0.43	0.686	238	0.76	0.482	15.2	1.08	0.329
%Acid × Time (*AC*)	−133	−0.42	0.692	8.9	0.55	0.604	505	1.61	0.168	5.3	0.37	0.724
Time × Power (*BC*)	−109	−0.34	0.745	25.7	1.61	0.169	−179	−0.57	0.594	−3.2	−0.23	0.829

**Table 4:** Analysis of variance (ANOVA) for the model evaluating lignans and tocopherol content under HCl and citric acid treatments.

**HCl** **Treatment**	**Source**	**Lignans**					**Tocopherol**				
		**DF**	**Adj SS**	**Adj MS**	**F-Value**	***p*-Value**	**DF**	**Adj SS**	**Adj MS**	**F-Value**	***p*-Value**
	Model	9	16968838	1885426	4.72	0.051	9	11525.4	1280.60	1.25	0.425
	Linear	3	10311812	3437271	8.61	0.020	3	4286.5	1428.85	1.39	0.348
	Square	3	5600277	1866759	4.68	0.065	3	4088.0	1362.67	1.33	0.364
	Interaction	3	1056750	352250	0.88	0.510	3	3150.8	1050.27	1.02	0.457
	Error	5	1996517	399303			5	5137.1	1027.42		
	Lack-of-Fit	3	1295305	431768	1.23	0.477 *	3	4480.4	1493.48	4.55	0.185 *
	Pure Error	2	701213	350606			2	656.7	328.33		
	Total	14	18965356				14	16662.5			
**Citric Acid Treatment**	**Source**	**Lignans**					**Tocopherol**				
		**DF**	**Adj SS**	**Adj MS**	**F-Value**	***p*-Value**	**DF**	**Adj SS**	**Adj MS**	**F-Value**	***p*-Value**
	Model	9	8743287	971476	2.46	0.167	9	10336.0	1148.45	1.45	0.355
	Linear	3	831917	277306	0.70	0.590	3	2176.8	725.60	0.92	0.496
	Square	3	6535365	2178455	5.53	0.048	3	7084.5	2361.50	2.99	0.135
	Interaction	3	1376006	458669	1.16	0.410	3	1074.7	358.24	0.45	0.726
	Error	5	1971320	394264			5	3950.2	790.05		
	Lack-of-Fit	3	1433210	477737	1.78	0.380 *	3	3573.3	1191.09	6.32	0.140 *
	Pure Error	2	538110	269055			2	377.0	188.48		
	Total	14	10714607				14	14286.3			

* Non-significant (*p*-value > 0.05) indicating the adequacy of the prediction model.

**Table 5:** The stability of the sesame oil samples.

Sesame Oil Samples	DPPH Activity (%)	TBARs (MDA kg^−1^)
Control	90.85 ± 0.03 ^e^	2.42 ± 0.02 ^c^
H15 (2% HCl: 540 W: 5 min)	95.08 ± 0.02 ^a^	2.90 ± 0.01 ^b^
H14 (10% HCl: 810 W: 10 min)	92.94 ± 0.01 ^d^	2.50 ± 0.03 ^c^
C1 (6% Citric acid: 810 W: 5 min)	94.47 ± 0.01 ^c^	2.13 ± 0.08 ^a^
C7 (10% Citric acid: 810 W: 10 min)	94.71 ± 0.01 ^b^	2.80 ± 0.01 ^b^

Means denoted by different letters indicate significant differences between treatments (*p* < 0.05). Means with the same letters indicate no significant difference between the values.

## Data Availability

The original contributions presented in the study are included in the article; further inquiries can be directed to the corresponding author.

## References

[1] Chavali S.R., Durga S.L. (2010). Sesame lignans and vitamin E potentiate the antihypertensive effect of losartan through modulation of vascular endothelial dysfunction. J. Med. Food.

[2] Namiki M. (2007). Nutraceutical functions of sesame: A review. Crit. Rev. Food Sci. Nutr..

[3] Pathak N., Rai A.K., Kumari R., Bhat K.V. (2020). Transformation of sesame oil into a functional ingredient: A review on recent trends in fortification. J. Food Sci. Technol..

[4] Mangialasche F., Solomon A., Kivipelto M., Mecocci P., Winblad B. (2010). Antioxidant vitamins and dementia: A systematic review. Alzheimer’s Dement..

[5] Alu’datt M.H., Rababah T., Alhamad M.N., Al-Mahasneh M.A., Almajwal A., Gammoh S. (2020). Effects of roasting temperature and time on the physicochemical properties of sesame (*Sesamum indicum* L.) seeds. J. Food Process. Pres..

[6] Sakar M., Akyüz K.C., Yılmaz L. (2016). Microwave and oven drying of honeydew melon: Thin layer drying behavior, modeling, quality, and energy aspects. J. Food Process Eng..

[7] Lu X., Wang M., Qi X., Fu Y., Shao Y., Zhou L., Xue S. (2018). Study on the microwave extraction process of lignans in sesame. Food Sci. Technol..

[8] Lee J.Y., Kim Y.S. (2014). Influence of roasting conditions on the physicochemical properties of sesame oil. Food Chem..

[9] Tareke E., Rydberg P., Karlsson P., Eriksson S., Törnqvist M. (2002). Analysis of acrylamide, a carcinogen formed in heated foodstuffs. J. Agric. Food Chem..

[10] Chen J., Chen Y.Z., Tian J.J., Ge H.F., Liang X.F., Xiao J.B., Lin H.T. (2018). Simultaneous determination of four sesame lignans and conversion in *Monascus* aged vinegar using HPLC method. Food Chem..

[11] Aoshima H., Yamaguchi M., Tani H. (2018). Acid-catalyzed epimerization of lignans: A mechanistic study. J. Org. Chem..

[12] Dai Q., Li Y., Wang M., Li Y., Li J. (2020). TlR2 and TlR4 are involved in the treatment of rheumatoid arthritis synovial fibroblasts with a medicated serum of asarinin through inhibition of Th1/Th17 cytokines. Exp. Ther. Med..

[13] Zhang W., Zhang J., Zhang M., Nie L. (2014). Protective effect of *Asarum* extract in rats with adjuvant arthritis. Exp. Ther. Med..

[14] Dai Q., Wang M., Li Y., Li J. (2019). Amelioration of CIA by asarinin is associated to a downregulation of TLR9/NF-κB and regulation of Th1/Th2/Treg expression. Biol. Pharm. Bull..

[15] Cai S.Q., Yu J., Wang X., Wang R.Q., Ran F.X., Shang M.Y., Namba T. (2008). Cytotoxic activity of some *Asarum* plants. Fitoterapia.

[16] Wang J., Qiu X., Li P., Hao D., Zhang M. (2018). Sesamin induces human hepatocellular carcinoma cell apoptosis via caspase activation and the ROS/JNK pathway. Arch. Pharm. Res..

[17] Xu Y., Zhang T., Xu L., Feng F. (2020). Structure and properties of structured lipids from enzymatic acidolysis of fish oil with oleic acid. Food Chem..

[18] Ferreira S.L., Bruns R.E., Ferreira H.S., Matos G.D., David J.M., Brandão G.C., dos Santos W.N. (2007). Box-Behnken design: An alternative for the optimization of analytical methods. Anal. Chim. Acta.

[19] Yuenyong J., Bennett C., Jiamyangyuen S., Mahatheeranont S., Sookwong P. (2024). Development of a simultaneous normal-phase HPLC analysis of lignans, tocopherols, phytosterols, and squalene in sesame oil samples. Foods.

[20] Yuenyong J., Pokkanta P., Phuangsaijai N., Kittiwachana S., Mahatheeranont S., Sookwong P. (2021). GC-MS and HPLC-DAD analysis of fatty acid profile and functional phytochemicals in fifty cold-pressed plant oils in Thailand. Heliyon.

[21] Blois M.S. (1958). Antioxidant determinations by the use of a stable free radical. Nature.

[22] Yamasaki K., Hashimoto A., Kokusenya Y., Miyamoto T., Sato T. (1994). Electrochemical method for estimating the antioxidative effects of methanol extracts of crude drugs. Chem. Pharm. Bull..

[23] Zeb A., Ullah F. (2016). A simple spectrophotometric method for the determination of thiobarbituric acid reactive substances in fried fast foods. J. Anal. Methods Chem..

[24] Coffigniez F., Briffaz A., Mestres C., Akissoé L., Bohuon P., El Maâtaoui M. (2019). Impact of soaking process on the microstructure of cowpea seeds in relation to solid losses and water absorption. Food Res. Int..

[25] Huang Y., Liu C., Ge Z., Huang F., Tang H., Zhou Q., Zheng C. (2023). Influence of different thermal treatment methods on the processing qualities of sesame seeds and cold-pressed oil. Food Chem..

[26] Muangrat R., Chalermchat Y., Pannasai S., Osiriphun S. (2020). Effect of roasting and vacuum microwave treatments on physicochemical and antioxidant properties of oil extracted from black sesame seeds. Curr. Res. Nutr. Food Sci..

[27] Ramroudi F., Yasini Ardakani S.A., Dehghani-Tafti A., Khalili Sadrabad E. (2022). Investigation of the physicochemical properties of vegetable oils blended with sesame oil and their oxidative stability during frying. Int. J. Food Sci..

[28] Ramroudi F. (2023). The antioxidant activity and oxidative stability of edible oils: A comparative study. Food Chem..

[29] Kamal-Eldin A. (1994). Lignan contents of sesame seeds and products. J. Agric. Food Chem..

[30] Zhao G., Wang H., Liu G., Wang Z.Q. (2016). Box-Behnken response surface design for the optimization of electrochemical detection of cadmium by square wave anodic stripping voltammetry on bismuth film/glassy carbon electrode. Sens. Actuators B Chem..

[31] Myers R.H., Montgomery D.C., Anderson-Cook C.M. (2016). Response Surface Methodology: Process and Product Optimization Using Designed Experiments.

[32] Mohamed Ahmed I.A., Musa Özcan M., Uslu N., Juhaimi F.A., Osman M.A., Alqah H.A., Babiker E.E. (2020). Effect of microwave roasting on color, total phenol, antioxidant activity, fatty acid composition, tocopherol, and chemical composition of sesame seed and oils obtained from different countries. J. Food Process. Preserv..

[33] Vaisali C., Charanyaa S., Belur P.D., Regupathi I. (2015). Refining of edible oils: A critical appraisal of current and potential technologies. Int. J. Food Sci..

[34] Čmolík J., Pokorný J. (2000). Physical refining of edible oils. Eur. J. Lipid Sci. Technol..

[35] Ansorena D., Ramírez R., Lopez de Cerain A., Azqueta A., Astiasaran I. (2023). Oxidative stability and genotoxic activity of vegetable oils subjected to accelerated oxidation and cooking conditions. Foods.

